# Temporal windows of reproductive opportunity reinforce species barriers in a marine broadcast spawning assemblage

**DOI:** 10.1038/srep29198

**Published:** 2016-07-04

**Authors:** Carla A. Monteiro, Cristina Paulino, Rita Jacinto, Ester A. Serrão, Gareth A. Pearson

**Affiliations:** 1 CCMAR, Universidade do Algarve, Campus de Gambelas, 8005–139 Faro, Portugal

## Abstract

Prezygotic isolating mechanisms act to limit hybridization and maintain the genetic identity of closely-related species. While synchronous intraspecific spawning is a common phenomenon amongst marine organisms and plays an important role in reproductive success, asynchronous spawning between potentially hybridizing lineages may also be important in maintaining species boundaries. We tested this hypothesis by comparing reproductive synchrony over daily to hourly timescales in a sympatric assemblage of intertidal fucoid algae containing selfing hermaphroditic (*Fucus spiralis* and *Fucus guiryi*) and dioecious (*Fucus vesiculosus* and *Fucus serratus*) species. Our results confirm that gametes are released on semi-lunar cycles in all species. However, sister species with different mating systems showed asynchronous spawning at finer circadian timescales, thus providing evidence for a partial reproductive barrier between hermaphroditic and dioecious species. Finally, our data also emphasize the ecological, developmental, and/or physiological constraints that operate to restrict reproduction to narrow temporal windows of opportunity in the intertidal zone and more generally the role of ecological factors in marine speciation.

Reproductive success in organisms with external fertilization is highly dependent on gamete encounter rates. Mechanisms such as spawning synchrony, optimal spawning conditions, morphological and physiological adaptations, and chemical signals (e.g., pheromone systems) all increase fertilization rates, particularly in sessile organisms^1^,^2^,^3^,^4^,^5^,^6^,^7^,^8^,^9^,^10^. The widespread occurrence of synchronous spawning amongst marine organisms suggests that the traits involved are strongly favoured by natural selection. However, where closely related species reproduce in sympatry, and where hybrids are less fit than the parental lineages, selection may also strongly favour asynchrony in reproductive timing between potentially hybridizing lineages. The evolution of such ecological mechanisms to minimize hybridization is crucial to preserve species identities, and may be key factors driving assortative mating during sympatric or ecological speciation^11^,^12^,^13^,^14^,^15^.

The brown algal genus *Fucus* (Phaeophyceae, Heterokontophyta) is a useful model for reproductive ecologists. Several species co-exist on North Atlantic rocky intertidal shores, occupying distinct but overlapping vertical niches with respect to tidal level and emersion stress intensity. Two major clades have been identified in *Fucus,* in both of which dioecious (outcrossing) and hermaphrodite (selfing) sister species have arisen^16^. Similar evolutionary patterns are observed in other groups of closely related plant and algal species, and is thought to promote reproductive isolation and divergence leading to speciation^17^,^18^,^19^,^20^, and to maintain species barriers^21^. Selfing increases reproductive assurance and colonizing capacity^22^,^23^, while reducing the chances of hybridization. However, the costs of inbreeding include lower genetic variation and effective population size compared with obligatory outcrossing dioecious species^24^,^25^. Hybridization in *Fucus* has been reported in several studies^19^,^26^,^27^,^28^,^29^,^30^,^31^,^32^,^33^, with hybrids reaching reproductive maturity in some cases^28^. Indeed, historical introgression has left clear evolutionary signals in extant lineages^33^. Despite this, contemporary levels of hybridization are apparently insufficient to blur the boundaries of distinct genetic entities in sympatry^29^,^34^.

The reproductive ecology of *Fucus* has been studied quite extensively; gametes are released with a semilunar periodicity and under calm water motion (i.e., following intervals of several hours under low current velocity, estimated as <0.2 m·s^−1^)^3^,^4^,^35^,^36^,^37^,^38^. However, comparative studies of spawning over fine circadian timescales are lacking for sympatric species assemblages. Such an approach might identify potential sources of reproductive isolation and improve our understanding of the evolution of reproductive isolation in marine broadcast spawners.

Here we present results of field and laboratory studies focusing on fine-scale temporal variation in gamete release during daily tidal cycles between two hermaphroditic and two dioecious *Fucus* congeners, to test whether differences in spawning time may act as a prezygotic barrier to hybridization between closely related species.

## Results

### Periodicity of egg release in nature

The peak of egg release in both hermaphroditic and dioecious species occurred with a periodicity of 2 weeks coincident with neap tides (Supplementary Fig. S1). Peaks of egg release were observed during all four neap tide periods monitored over 2 months, and the majority of days on which release occurred were coincident across all species.

**Figure 1:**
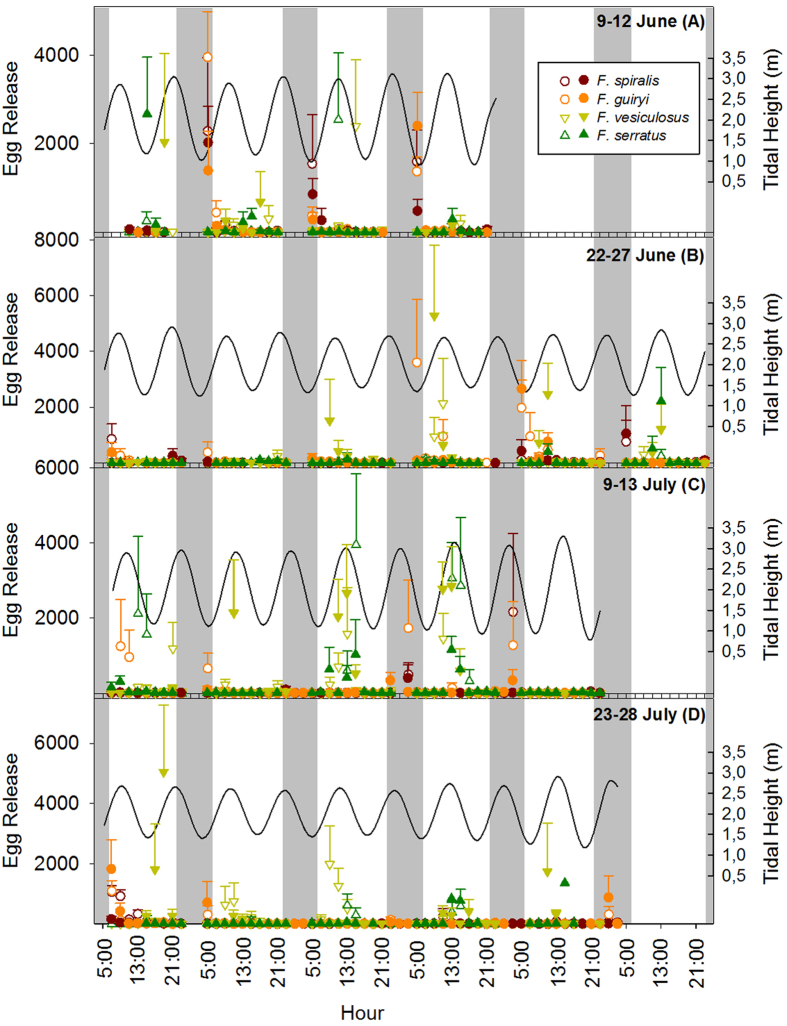
Egg release during 2 h sampling intervals (n = 5 ± SE) by hermaphroditic (circles) *Fucus spiralis*, *Fucus guiryi*, and dioecious (triangles) *Fucus vesiculosus* and *Fucus serratus* at two replicate sites (open and closed symbols) during four neap tide periods. Black lines show tidal heights and grey bars the night periods.

During the 4 neap tide periods studied in 2011 (Fig. 1), major egg release events (defined as >1000 eggs per bag in a 2 h sampling period) were observed on 7 days in *F. spiralis* (June 10–12, 27 and July 13, 22–23); 9 days in *F. guiryi* (June 10, 12, 25, 26 and July 9, 12, 13, 23, 26), 15 days in *F. vesiculosus* (June 9, 13, 25–27 and July 9, 25, 27) and 7 days in *F. serratus* (June 9, 11, 27 and July 9, 11, 12, 27). While gamete release co-occurred in all four species on several days and mostly when individuals were immersed, the timing of spawning within the tidal cycle was clearly divergent between dioecious (*F. vesiculosus* and *F. serratus*) and hermaphroditic (*F. spiralis* and *F. guiryi*) species (Fig. 1).

**Figure 2:**
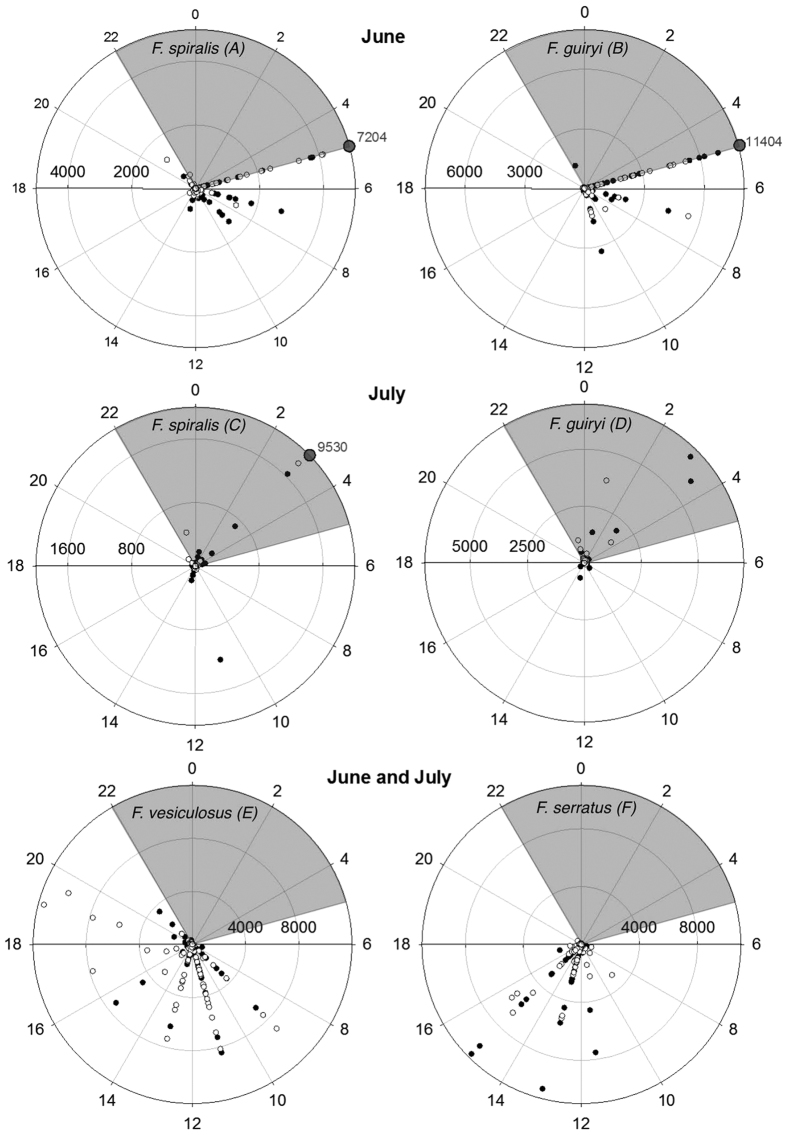
Egg release (radial data) relative to timing of day (angular data) by (**A**) *Fucus spiralis*, (**B**) *Fucus guiryi*, in June (sampling between 05:00 and 22:00 h); (**C**) *Fucus spiralis*, (**D**) *Fucus guiryi* in July; (**E**) *Fucus vesiculosus* and (**F**) *Fucus serratus* in June and July at two replicate sites (open and closed symbols). Grey boxes are the dark periods during daily cycle.

**Figure 3:**
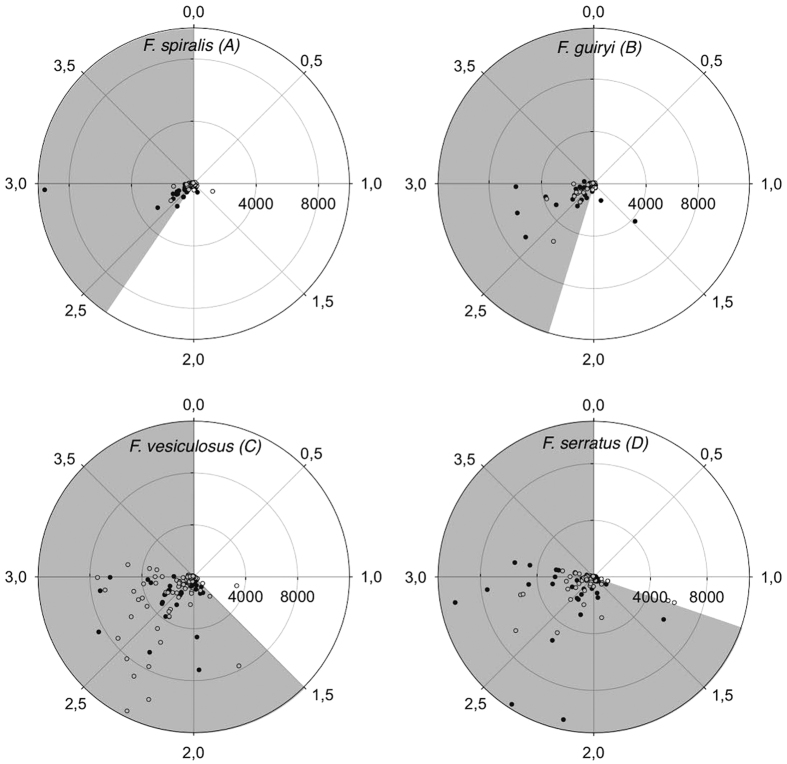
Egg release (radial data) relative to tidal level (angular data) by (**A**) *Fucus spiralis*, (**B**) *Fucus guiryi*, (**C**) *Fucus vesiculosus* and (**D**) *Fucus serratus* at two replicate sites (open and closed symbols). Grey boxes indicate periods of immersion during the tidal cycles.

Since hermaphroditic species consistently released eggs within the period 22:00–05:00 h in June (Fig. 1A,B), in July we sampled egg release during the night between Jul 10–13 and 24–28 (Fig. 1C,D). This sampling confirmed that spawning was coincident with the nightly high tide (Fig. 1C,D). In *F. spiralis* the major egg release event was observed at 03:00 h on 13 July (Figs 1C and 2C) and in *F. guiryi* maximum release was recorded at 01:00 and 03:00 h on July 12–13 and 28 (Figs 1C,D and 2D). Some early morning release was also seen coinciding with the early high tides (Fig. 1A–D). In both species peaks of egg release (≥200 eggs per bag) frequently occurred when individuals were immersed (Fig. 3A,B).

In contrast to hermaphrodites, both dioecious species released eggs exclusively during the daytime (Figs 1A–D and 2E,F). The majority of release in *F. serratus* took place between 11:00 and 15:00 h. Timing appeared somewhat less tightly constrained in *F. vesiculosus* (c.f. Fig. 2E,F), but major egg release events clustered mainly around peak daytime high tides for both species (Figs 1 and 3C,D).

### Experimental manipulation of spawning conditions

The comparison of egg release by *F. guiryi* (hermaphroditic) and *F. vesiculosus* (female dioecious) under experimentally manipulated tidal (high versus low tide) and circadian (light versus dark) regimes showed that these two species differed in their patterns of cumulative egg release over superimposed circadian and tidal cycles (Table 1A; Sp x Ti(Ta) x Sa interaction). However, tidal cycle phase (tank 1 versus tank 2) had no effect, indicating that egg release was entrained more by environmental conditions rather than intrinsic rhythms.

**Figure 4:**
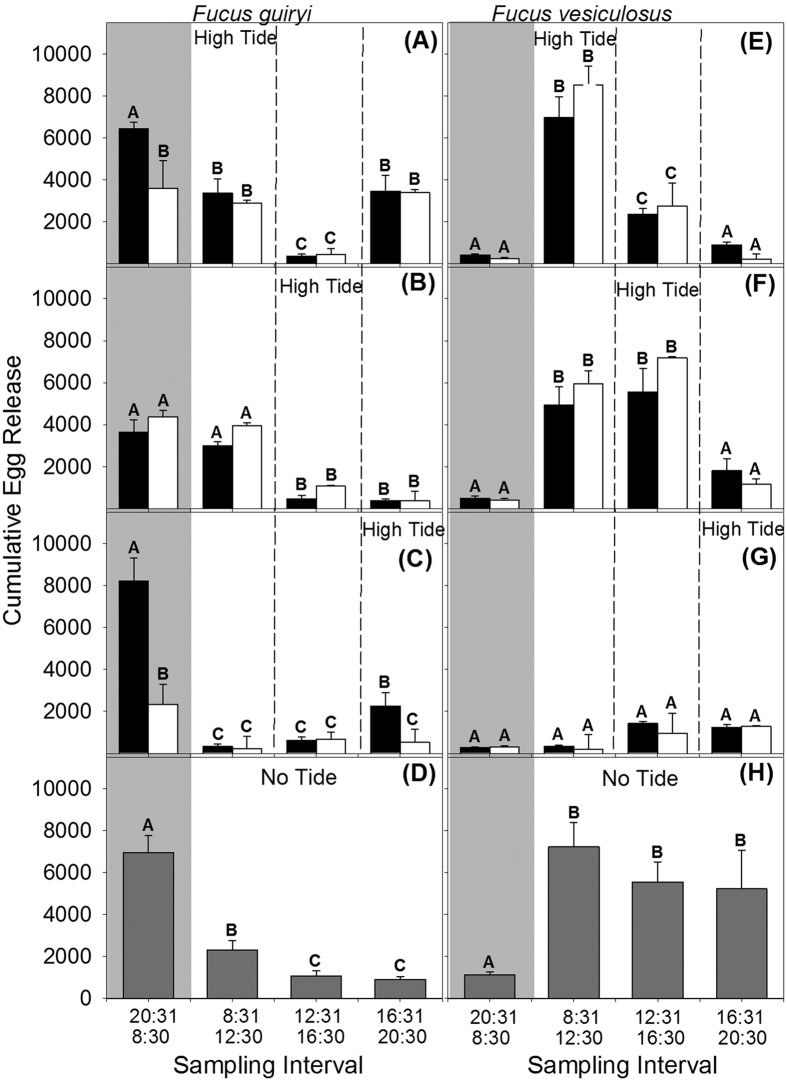
Effects of tidal shifts and circadian cycle on egg release by *Fucus guiryi* and *Fucus vesiculosus*. Cumulative egg release (n = 8 ± SE) under (**A**,**E**) high tide between 8:00 and 12:00 h; (**B**,**F**) high tide between 12:01 and 16:00 h; (**C**,**G**) high tide between 16:01 and 20:00 h; and (**D**,**H**) constant immersion (no tidal regime). Black and white bars represent the means in each tank. Dark grey bars represent the means in tank with no tidal regime. Different letters above bars indicate significant differences (PERMANOVA, p < 0.05). Light grey shading indicates the night periods and longitudinal lines (black dotted lines) separate the timing of high and low tide periods.

In hermaphroditic *F. guiryi*, egg release consistently occurred during the night (20:31–08:30 h) in all tanks, and extended into the morning period (08:30–12:30) when the high tide occurred in the morning (08:31–12:30 h), or early afternoon (12:31–16:30 h) (Fig. 4A,B). During earlier high tides (08:31–12:30 h) a second late afternoon peak of egg release was observed (16:30–20:30 h; Fig. 4A). When the high tide was later (16:31–20:30 h) egg release was more restricted to the dark period (Fig. 4C). Egg release was lowest in the middle of the day (12:31–16:30 h), irrespective of the tidal cycle.

In contrast, egg release was very low during the night in dioecious *F. vesiculosus*, irrespective of the tidal cycle conditions (Fig. 4E–G). We observed significant peaks of release corresponding with high tide (08:31–12:30 h; Fig. 4E), and prior to and during high tide when high tide is later (12:31–16:00 h; Fig. 4F). In contrast, when the high tide was in the late afternoon, very little egg release was observed (16:31–20:30 h; Fig. 4G), although significantly more eggs were counted at 16:30 and 20:30 h than at earlier sampling times.

In the absence of tides, a significant interaction (Table 1B) was observed between species and sampling interval. While under constant immersion the greater amount of egg release in *F. guiryi* occurred during the dark period, in *F. vesiculosus* egg release occurred throughout the day, with no significant difference between daytime sampling intervals (Fig. 4D,H).

**Figure 5:**
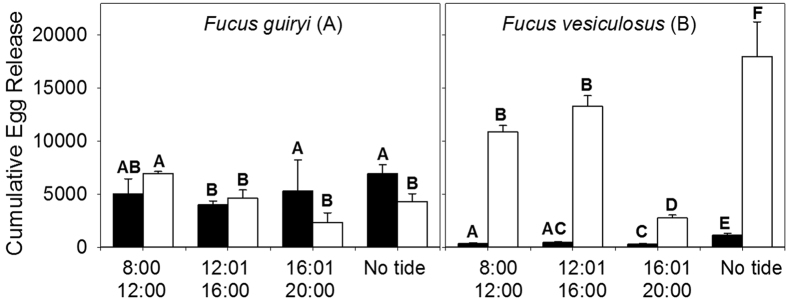
Effects of tidal shifts and circadian cycle on egg release by *Fucus guiryi* and *Fucus vesiculosus*. Cumulative egg release (n = 8 ± SE) under high tide between 8:00–12:00 h, 12:01–16:00 h, 16:01–20:00 h and constant immersion (no tidal). Black and white bars represent the means of egg release at night and day, respectively. Different letters above bars indicate significant differences (PERMANOVA, p < 0.05).

For circadian cycles (day and night), no significant differences were observed between the numbers of eggs released by *F. guiryi* between night and day when the high tide was between 8:00–12:00 h and 12:01–16:00 h (Fig. 5A and Table 1C). However, significant differences were observed for high tides later in the day (16:01–20:00 h) and for atidal conditions; in both cases the amount of egg release in *F. guiryi* was higher at night than during the day. In contrast, egg release in *F. vesiculosus* was always significantly higher during the day than at night (Fig. 5B and Table 1C).

## Discussion

The experimental and field data presented in this study provides clear evidence for divergent reproductive timing between congeners in an assemblage of intertidal fucoid algae. The differences we found in spawning time have evolved recently, alongside variation in reproductive mode and mating system^16^. While at semilunar timescales the four congeners studied share a common spawning pattern in northern Portugal, the previously unrecognised divergence in spawning times during circadian cycles supports the hypothesis that temporal (partial) reproductive isolation has evolved. At least under these ecological conditions, this timing divergence might constitute an ecological barrier to hybridization within the most closely related members of the *F. vesiculosus* subclade.

Reproduction is highly constrained by environmental cycles in the intertidal; the interaction of tidal (immersion-emersion) and circadian light-dark cycles are crucial cues that regulate spawning in fucoids^3^,^36^,^37^,^38^,^39^. Our data confirm previous reports^36^ that natural gamete release occurs preferentially during high tide immersion. However, we found that while dioecious *F. vesiculosus* and *F. serratus* spawned during daytime neap high tides, two hermaphroditic species sister to *F. vesiculosus* spawned mainly during night-time high tides during the same phase of the semilunar cycle, a pattern that has not been observed previously^3^. The divergence in circadian patterns of spawning between hermaphroditic (*F. spiralis* and *F. guiryi*) and dioecious (*F. vesiculosus*) sister species is striking given their divergence time may be less than 1 MYA^16^. Earlier-diverging dioecious members of the genus all share a pattern of daytime high-tide spawning^35^,^36^,^37^, which therefore appears to be the ancestral state within *Fucus*, while nocturnal/early morning spawning in the hermaphrodites *F. guiryi* and *F. spiralis* indicates a recent change to a modified or alternate signal – response pathway. Gamete release in fucoids involves a water-motion sensing system based on photosynthetic carbon acquisition^4^,^38^, linked by downstream signalling to turgor changes that are presumed to directly trigger the expulsion of gametes^40^,^41^. Nocturnal spawning has presumably arisen either by bypassing the photosynthesis dependent part of the process, or to modifications in timing of subsequent parts of the pathway.

Whatever the mechanism(s) involved, the potential ecological drivers of nocturnal spawning patterns may be linked with habitat, as both *F. guiryi* and especially *F. spiralis* are stress-tolerant species with vertical ranges that extend higher than either low-mid intertidal *F. serratus* or mid-intertidal *F. vesiculosus. F. spiralis* inhabits the upper intertidal zone, and even during high tide this species may be under water for less than 30 min, while during extreme neap tides individuals remain uncovered at high tide. Therefore, escape from desiccation, thermal and/or irradiance stress on eggs, sperm and embryos and selection for recruitment success may be a driver of nocturnal/early morning spawning.

Laboratory experiments in which only tidal and circadian cycles were manipulated were able to capture much of the complexity of natural spawning rhythms (Fig. 4), confirming differential spawning patterns between *F. guiryi* (hermaphroditic) and *F. vesiculosus* (dioecious), in broad agreement with field observations. Indeed, simple light:dark cycling without tidal treatment was sufficient to produce hermaphrodite – dioecious (nocturnal – diurnal) spawning patterns (Figs 4 and 5). Spawning was also qualitatively unaffected by changing the tidal phase in experimental tanks, indicating that any potential intrinsic rhythms are secondary to the proximal environmental cues that trigger gamete release. Spawning was suppressed during darkness in *F. vesiculosus*, and was dependent on timing of high tides in the light. In contrast, cumulative spawning in cultured *F. guiryi* was similar or greater in darkness than in the light, independent of the timing or presence of tides (Figs 4 and 5). The main difference between field and culture conditions was the tendency for *F. guiryi* to spawn late in the day in culture prior to the night-time high tide (Fig. 4A), perhaps a consequence of relaxed stress regime with no desiccation and moderate temperature (14 °C).

Some early morning spawning events in natural stands of *F. spiralis* and *F. guiryi* occasionally overlapped with dioecious species, particularly earlier in the reproductive season (June; Fig. 1A,B). Thus, temporal segregation of spawning at the interspecific level within the assemblage is incomplete, and the ecological conditions for hybridization exist between all four species, which coexist within a few meters of each other on the shore. Despite early reports of high levels of hybrid fertility between *F. vesiculosus* and *F. serratus*^42^, compelling experimental evidence for strong (although incomplete) prezygotic barriers were later reported^43^. In contrast, early reports as well as more recent molecular evidence support the occurrence of hybridization within both the *F. vesiculosus*^19^,^28^,^29^,^34^, and *F. serratus* subclades^32^. In potentially hybridizing lineages, ecological barriers such as temporal variation in reproduction may be strongly selected traits, as seems to be the case here. The main examples for marine broadcast spawners have been reported for corals: small temporal differences in gamete release of ca. one hour were observed between corals within the genus *Montastraea*^15^, and small variations have been observed in other sympatric coral species^1^,^15^,^44^,^45^,^46^. Interestingly, as we observed here in *Fucus*, an inverse relationship between interspecific spawning synchrony and phylogenetic distance has been seen in *Montastraea*^15^.

Phylogenetic divergence and build-up of gametic incompatibility can explain why spawning times can overlap in sympatric populations of dioecious *Fucus* species without risk of excessive hybridization. Other ecological mechanisms, such as release of eggs in high concentrations of mucilage (pers. obs.) may also play a role in limiting the dispersal of gametes^6^,^47^. However, given the highly coincident spawning between *F. spiralis* and *F. guiryi*, what prevents hybridization between these sister species? The answer appears to be that a shift in reproductive mode to hermaphroditism, together with a predominantly selfing mating system is sufficient^19^,^20^,^48^. It may help that hermaphrodites produce relatively little sperm^28^, which is released simultaneously from the same reproductive structures (receptacles) as the eggs.

The relative contributions of pre- and post-zygotic barriers to the evolutionary history of the genus *Fucus* are unknown. Several sources of evidence support both hypotheses of pre- and post-zygotic barriers as important in our study species. First, the occurrence of a range of intermediate genotypes in the field^19^,^29^, indicates that hybrids and introgressed individuals can be reproductively viable, lacking intrinsic complete post-zygotic barriers. However, comparative hybrid fitness studies are lacking. Second, the rarity of such hybrids in the field (see references above) and the persistence of each species as cohesive genetic entities, indicates that although hybrids can be viable, they are rarely produced (prezygotic barriers), are less fit (post-zygotic barriers), or likely both. The observation that hybrids are rare outside of contact zones matches both of the previous hypotheses. Our study demonstrates that reproductive ecology effectively acts as a prezygotic barrier for some species, but does not claim that it is the only barrier, and indeed it cannot be for species with similar mating systems. In addition, there might also be a role of partial gamete compatibility in mediating such barriers, allowing only some rare hybrid matings, but further work is necessary to assess this hypothesis.

Our study shows that spawning synchrony (constraints) on semilunar timescales within an intertidal assemblage masks spawning asynchrony on smaller time scales (circadian and tidal cycles) in interfertile sister species of fucoid seaweeds. This likely represents an early-evolving and critical ecological mechanism that reinforces prezygotic isolation and maintains species boundaries between sister taxa of these externally-fertilizing broadcast spawners. Where interspecific spawning is synchronous, evidence from the literature suggest that phylogenetic distance is sufficient to prevent frequent crossing^43^, while genetic data suggest that mating system is an additional prezygotic mechanism against hybridization by minimizing gene flow between selfing hermaphrodites^20^. The cues that trigger spawning during tidal immersion in all species are generated by the combined effects of circadian and tidal cycles. However, further studies, perhaps genome-enabled analyses, will be required to understand the mechanisms underlying the recent evolutionary shift between diurnal and nocturnal spawning patterns described here.

## Material and Methods

### Study site and species

The study site was Viana do Castelo, northern Portugal (41°41′47N 8°51′10W), which is the southernmost sympatric distributional limit of the four species of *Fucus* studied. There, *F. spiralis* is found in the high intertidal zone; *F. guiryi* and *F. vesiculosus* in mid-intertidal zone; *F. serratus* in the low-intertidal zone.

The gametes in all species of the genus *Fucus* develop inside gametangia in specialized apical structures called receptacles. In dioecious species, the sperm and eggs develop in different individuals (male and female) whereas in hermaphrodites both egg and sperm occur in same individual. Spawning consists in the release of gametangia that are negatively buoyant (i.e., they sink). Each female gametangium (oogonium) contains 8 eggs (non motile, ca. 80 μm in diameter) and each male gametangium (antheridium) contains 64 sperm (motile, ca. 5 μm in length). The gametangia open shortly upon release in seawater liberating negatively bouyant eggs and negatively phototactic sperm (which therefore swim towards the bottom). Fertilization then occurs externally, and most likely near the substrate. Fertilization success in *Fucus* species has been shown to be high^3^,^4^,^35^,^36^,^37^,^38^. Egg dispersal is highly restricted since eggs tend to fall immediately below the releasing individual^49^,^50^. The occurrence of fertilization shortly after synchronous egg and sperm release together with low gamete dispersal might function as partial prezygotic barriers preventing hybridization between species occupying different tidal zones. Putative hybrids (identified as intermediate genotypes) were found mainly in the contact zones where species overlap, however they are rare^19^,^29^.

Mature reproductive individuals of *F. guiryi* (hermaphroditic) and female *F. vesiculosus* (dioecious) were collected from the same site for tidal and circadian laboratory experiments. Species were identified as described previously^34^. Sampling of eggs (for natural spawning patterns) and mature individuals (for experimental manipulation of spawning conditions) took place in the middle of their respective intertidal range, to avoid hybrids that are mainly found at overlapping range edges^19^,^29^.

### Natural spawning patterns – semilunar and tidal timescales

Egg release at semilunar timescales was estimated using rugose artificial substrates (5.96 cm^−2^) to retain settled eggs. Egg settlement for the 4 species was monitored daily at two sites between Jun 7 and Aug 3, using five disks per site per species fixed under the algal canopy, as described previously^37^,^39^.

Egg release during tidal cycles was monitored during four periods, consisting of a few days before and after the neap tides (lower tidal amplitude), when spawning peaks take place^37^. These were the days when minimal low tide level was higher than ca. 1 m and the maximal high tide level was lower than ca. 3 m, in Jun (9–12 and 22–27) and Jul (9–13 and 23–28). Nylon mesh bags (40 μm) were used to retain eggs; *Fucus* eggs are all larger than 60 μm^51^. Each bag contained 2–3 receptacles per individual (females for dioecious species). During each sampling period, for each species, 5 individuals (1 bag per individual) were monitored for egg release at each of 2 sites (separated by approximately 5 m)^37^. The bags were collected and replaced every 2 h between 5:00 and 22:00 h in June 9–12 and 22–27 and July 9 and 23 (the first and last samples were taken in darkness). To complement the data with detailed patterns of night release, in July the sampling period was extended over the night, i.e., over 24 h per day (sampling was performed every 2 h during 88 and 94 consecutive hours in Jul 10–13 and 24–28, respectively).

### Experimental manipulation of spawning conditions

The effects of light and tidal cycles on the timing of gamete (egg) release were studied in *F. guiryi* and *F. vesiculosus* in a laboratory experiment. *F. vesiculosus* (dioecious) was sexed in the laboratory to select females; hermaphroditic receptacles (*F. guiryi*) contain both oogonia and antheridia. Mature receptacles were excised and acclimated in individual 50 mL tubes (Falcon) containing 40 mL filtered seawater (SW; 35 psu) for 2 days prior to quantification of egg release, and SW was replaced daily.

In a culture chamber (14 °C; 12:12 h light-dark cycle; 100 μmol photons m^−2^s^−1^), tidal regimes were simulated in tanks for 24 days as follows: Tank 1 – timing of high and low tide coincident with that at Viana do Castelo. Tank 2 – opposite phase to tank 1, i.e., peak low tide in tank 2 corresponded to peak high tide in tank 1. Tank 3 – no tides, receptacles were constantly immersed. Tides were programmed by timers controlling the pumping and draining of SW in the tanks (complete pumping and draining each took ca. 5 min). Receptacles were submerged for 4 h per high tide, corresponding to 2 h on either side of the natural timing of high tide (tank 1) or of low tide (tank 2) in the field. Immersion time was within the range seen by both species on the shore. Eight individuals were used as replicates for each species. For each species and tank, two receptacles of similar size were placed in each of n = 8 tubes. To allow SW to drain at low tide a small hole was made in the base of the tubes, protected by nylon mesh (40 μm) to retain the eggs. Egg release was quantified for 24 days, receptacles were transferred to tubes with fresh SW at 8:30 h, 12:30 h, 16:30 h and 20:30 h (no collection was performed at night). The eggs present in each tube were counted under a dissecting microscope. The numbers of eggs released were comparable across replicates within species on the basis of equal amounts of reproductive tissue (2 receptacles) being used per replicate. However, fecundity was not tested in this study because the variable of interest was the timing of maximum gamete release, rather than absolute numbers of gametes released, to assess our hypothesis (i.e., whether differences in spawning time may act as a prezygotic barrier to hybridization between closely related species). Previous studies^28^,^37^ have shown that the variability in the amounts of eggs produced is orders of magnitude lower than the variability between the numbers of eggs released on a peak spawning day versus the amounts released on other days.

### Experimental manipulation of spawning conditions – statistical analyses

Analyses aimed to test both effects of circadian and tidal regimes. Cumulative egg release in tidal shift treatments (tidal conditions; tank 1 and 2) was analyzed under the following design: species (2 levels: *F. guiryi* and *F. vesiculosus*, orthogonal and fixed), tanks (2 levels, orthogonal and fixed), daytime high tide interval (3 levels: between 8:00–12:00 h, 12:01–16:00 h and 16:01–20:00 h, nested within tanks) and sampling time (4 levels: 8:30 h, 12:30 h, 16:30 h and 20:30 h, orthogonal and fixed).

To assess the effects of circadian light:dark intervals on egg release by *Fucus* in the absence of tides, cumulative egg release in the tank without tides (atidal condition) was analyzed under the following design: species (2 levels: *F. guiryi* and *F. vesiculosus*, orthogonal and fixed) and sampling timing (4 levels: 8:30 h, 12:30 h, 16:30 h and 20:30 h, orthogonal and fixed).

To test for differences in cumulative egg release between light and dark periods (circadian cycles) the following design was analyzed: species (2 levels: *F. guiryi* and *F. vesiculosus*, orthogonal and fixed), tide conditions (4 levels: 8:00–12:00 h, 12:01–16:00 h, 16:01–20:00 h and no tide, orthogonal and fixed) and circadian cycles (2 levels: day and night, orthogonal and fixed).

In all analyses the number of replicates was eight and cumulative egg release for each sampling interval was summed over 24 days. Means were compared using PERMANOVA^52^. The permuted *p*-value was the number of times the *p*-value was equal to or outside the 95% confidence interval divided by the total number of permutations (9999).

## Additional Information

**How to cite this article**: Monteiro, C. A. *et al*. Temporal windows of reproductive opportunity reinforce species barriers in a marine broadcast spawning assemblage. *Sci. Rep.* **6**, 29198; doi: 10.1038/srep29198 (2016).

## Supplementary Material

Supplementary Information

## Figures and Tables

**Table 1:** Results of PERMANOVAs testing the cumulative egg release in tidal shifts, in constant immersion (atidal conditions) and at circadian cycles.

	Source of variance	df	F	*P*
**Tidal condition (A)**	Sp	1	0.0014	0.9738
	Ta	1	0.1088	0.8951
	Ti (Ta)	4	22.5528	0.0001*
	Sa	3	1.8161	0.1964
	Sp x Ta	1	0.7522	0.4269
	Sp x Ti (Ta)	4	10.6207	0.0001*
	Sp x Sa	3	14.1226	0.0004*
	Ta x Sa	3	0.4804	0.6953
	Ti (Ta) x Sa	12	14.4957	0.0001*
	Sp x Ta x Sa	3	0.4235	0.7485
	Sp x Ti (Ta) x Sa	12	7.0750	0.0001*
	Residual	336		
**Atidal condition (B)**	Sp	1	9.4194	0.0023*
	Sa	3	1.4363	0.2346
	Sp x Sa	3	16.484	0.0001*
	Residual	56		
**Circadian Cycles (C)**	Sp	1	22.4222	0.0001*
	Ti^	3	10.2701	0.0001*
	Ci	1	23.8428	0.0001*
	Sp x Ti^	3	16.1779	0.0001*
	Sp x Ci	1	75.0505	0.0001*
	Ti^ x Ci	3	19.4517	0.0001*
	Sp x T^ x Ci	3	4.3833	0.0040*
	Residual	112		

Sp; species (*Fucus guiryi* and *Fucus vesiculosus*), Ta; tanks (tidal phase coincident or opposite that at the collection site in the field); Ti; tides (high tide between 8:00–12:00 h, 12:01–16:00 h and 16:01–20:00 h) and Sa; sampling time (8:30 h, 12:30 h, 16:30 h and 20:30 h), Ti^; tides (high tide between 8:00–12:00 h, 12:01–16:00 h, 16:01–20:00 h and no tidal) and Ci; circadian cycles (night and day sampling). Significant differences (P < 0.05) are noted with*.
