# Supplementary material for: Temporal windows of reproductive opportunity reinforce species barriers in a marine broadcast spawning assemblage
Source: Sci Rep. 2016 Jul 4;6:29198. doi: 10.1038/srep29198 (PMC4931575; doi:10.1038/srep29198)
Supplement: Supplementary Information [file srep29198-s1.pdf]

Supplementary Information

Temporal windows of reproductive opportunity reinforce species barriers in a marine broadcast spawning assemblage

Carla A. Monteiro, Cristina Paulino, Rita Jacinto, Ester A. Serrão, Gareth A. Pearson

Supplementary Figure

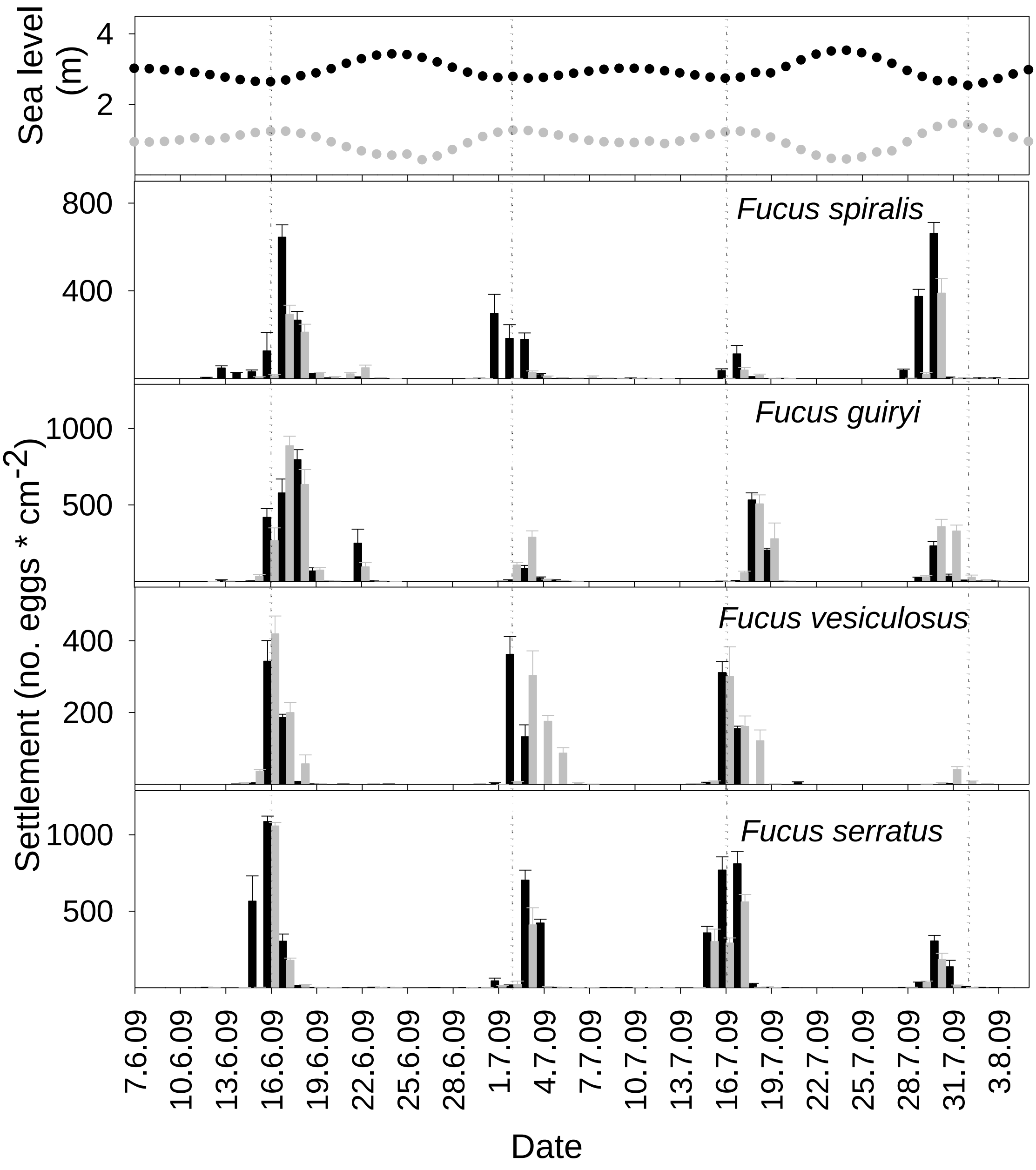

Figure S1. Daily egg settlement (n = 5 ± SE) from *Fucus spiralis, Fucus guiryi*, *Fucus vesiculosus* and *Fucus serratus*, at two replicate sites (dark bars and grey bars) between 7 June and 5 August 2009. The low tide (open symbols) and high tide (closed symbols) levels are shown in the first panel. Vertical black dotted lines indicate the peak of neap tides. Settlement data for *Fucus guiryi* and *Fucus vesiculosus* was published in Monteiro et al[S1].

Supplementary Reference

S1. Monteiro, C. A., Serrão, E. A. & Pearson, G. A. Reproductive investment, synchrony and recruitment success in marine broadcast spawners: effects of mating system and habitat (exposed shore versus estuary). Mar. Environ. Res. **112**, 33-39 (2015).
